# Aluminum nitride nanophotonics for beyond-octave soliton microcomb generation and self-referencing

**DOI:** 10.1038/s41467-021-25751-9

**Published:** 2021-09-14

**Authors:** Xianwen Liu, Zheng Gong, Alexander W. Bruch, Joshua B. Surya, Juanjuan Lu, Hong X. Tang

**Affiliations:** 1 Department of Electrical Engineering, Yale University, New Haven, CT 06511 USA; 2 Present Address: School of Optics and Photonics, Beijing Institute of Technology, Beijing, China

**Keywords:** Nonlinear optics, Solitons, Frequency combs, Optical metrology

## Abstract

Frequency microcombs, alternative to mode-locked laser and fiber combs, enable miniature rulers of light for applications including precision metrology, molecular fingerprinting and exoplanet discoveries. To enable frequency ruling functions, microcombs must be stabilized by locking their carrier-envelope offset frequency. So far, the microcomb stabilization remains compounded by the elaborate optics external to the chip, thus evading its scaling benefit. To address this challenge, here we demonstrate a nanophotonic chip solution based on aluminum nitride thin films, which simultaneously offer optical Kerr nonlinearity for generating octave soliton combs and quadratic nonlinearity for enabling heterodyne detection of the offset frequency. The agile dispersion control of crystalline aluminum nitride photonics permits high-fidelity generation of solitons with features including 1.5-octave spectral span, dual dispersive waves, and sub-terahertz repetition rates down to 220 gigahertz. These attractive characteristics, aided by on-chip phase-matched aluminum nitride waveguides, allow the full determination of the offset frequency. Our proof-of-principle demonstration represents an important milestone towards fully integrated self-locked microcombs for portable optical atomic clocks and frequency synthesizers.

## Abstract (web summary)

Though octave soliton microcombs are attractive for on-chip metrology and optical clocks, limitations in existing materials lead to increased chip integration complexity. Here, the authors report access to octave soliton microcombs and self-referencing using aluminium nitride nanophotonic chips.

## Introduction

Optical frequency combs, developed from solid-state or fiber mode-locked lasers, have evolved into photonic chip-based sources that feature the potential towards a miniaturized footprint and reduced cost^1^. Among various chip-scale schemes^2–5^, microresonator Kerr frequency combs (“microcombs” hereafter) are of particular interest because of their high scalability for photonic integration^6–8^. To enable phase-coherent microcombs, substantial efforts have been made towards soliton mode-locking on the one hand^9–17^, unveiling rich soliton physics on the other hand^18–20^. Specifically, octave-spanning soliton microcombs are important because it permits phase locking of the carrier-envelope offset (CEO) frequency (*f*_ceo_) via well-known *f*–2*f* interferometry^21^, and are prerequisite for chip-scale implementation of precision metrology^22^, frequency synthesizers^23^ and optical clocks^24^. To date, silicon nitride (Si_3_N_4_) nanophotonics has proved viable for octave soliton operations with a terahertz repetition rate (*f*_rep_)^25–27^. Nevertheless, such a large *f*_rep_ is not amenable for direct photodetection and poses challenges to access the CEO frequency with a value up to *f*_rep_. In the meantime, the lack of intrinsic quadratic *χ*^(2)^ nonlinearities in Si_3_N_4_ films typically requires an external frequency doubler and off-chip optical circuitry for deriving the CEO frequency^28,29^. These off-chip optical components compromise the scaling advantage of microcombs and significantly set back self-locked microcombs for portable applications.

Aluminum nitride (AlN) semiconductors exhibit a non-centrosymmetric crystal structure, thereby possessing inherent optical *χ*^(2)^ nonlinearity as well as Pockels electro-optic and piezoelectric properties^30^. Apart from the advances in ultraviolet light-emitting diodes^31^ and quantum emitters^32,33^, AlN has also proved viable for low-loss nanophotonics in high-efficiency second-harmonic generation (SHG)^34,35^ and high-fidelity Kerr and Pockels soliton mode-locking^15,36^. Therefore, it is feasible to establish an on-chip *f*–2*f* interferometer provided that an octave AlN soliton microcomb is available. This is a solution that is favored here comparing with the heterogeneous integration approach such as proposal based on hybrid gallium arsenide (GaAs)/Si_3_N_4_ waveguides^37^. Despite that on-chip *f*_ceo_ detection was achieved from supercontinuua driven by a femtosecond laser in non-resonant *χ*^(2)^ nanophotonic waveguides made from AlN^38^ or lithium niobate (LN) thin films^39,40^, resonator microcomb-based *f*–2*f* interferometry using nanophotonics, to our knowledge, remains elusive.

In this article, we demonstrate high-fidelity generation of octave soliton microcombs and subsequent *f*_ceo_ detection using AlN-based nanophotoinc chips. Thanks to mature epitaxial growth, AlN thin films with highly uniform thickness are available, thus permiting lithographic control of group velocity dispersion (GVD) for comb spectral extension via dispersive wave (DW) emissions^11^. Our octave soliton microcombs possess separated dual DWs and moderate *f*_rep_ of 433, 360, and 220 gigahertz, and are found to be reproducible from batch-to-batch fabrications. The results then allow us to capture the *f*–2*f* beatnote through on-chip SHG in phase-matched AlN waveguides. Our work establishes the great potential of non-centrosymmetric AlN photonic platforms for achieving portable self-locked microcomb sources in the near future.

## Results

### Experimental scheme description

**Fig. 1:**
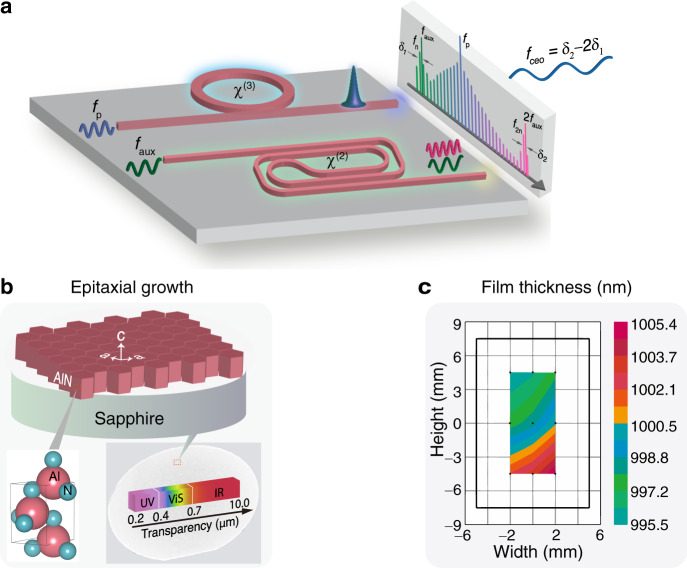
Experimental scheme. **a** Illustration of *f*–2*f* interferometry using octave-spanning soliton microcombs and second-harmonic generators in a nanophotonic platform harboring both *χ*^(3)^ and *χ*^(2)^ susceptibilities. The offset frequency *f*_ceo_ is accessible from the beatnotes of *δ*_1_ and *δ*_2_, and *f*_p_ is the pump laser frequency. **b** Top: sketch of a hexagonal aluminum nitride (AlN) layer (lattice constants: *a* and *c*) epitaxially grown on a *c*-plane sapphire substrate. Bottom: unit cell of an AlN crystal (left) and photograph image of a 2-inch AlN wafer featuring a broad transparency window from ultraviolet (UV), visible (ViS) to infrared (IR) regimes (right). **c** Spectroscopic ellipsometer mapping of the AlN film thickness in a region of 4 × 9 mm^2^, showing a minor variation of 1000 ± 5 nm denoted by the right color bar.

Figure 1a illustrates the implementation of microcomb-based *f*–2*f* interferometry from a nanophotonic chip. The strategy is to leverage non-centrosymmetric photonic media for co-integration of *χ*^(3)^ octave soliton microcombs and *χ*^(2)^ SHG doublers. For a proof-of-principle demonstration, we adopt an auxiliary laser (at *f*_aux_) to obtain sufficient SHG power (at 2*f*_aux_) from phase-matched optical waveguides. The use of the auxiliary laser can be eliminated by exploiting microring-based architectures to boost the SHG efficiency^34^. By subsequently beating *f*_aux_ and 2*f*_aux_ with the *f*_n_ and *f*_2n_ comb lines at their corresponding beatnotes of *δ*_1_ and *δ*_2_, the *f*_ceo_ signal reads:

1 $${f}_{{{{{{{{\rm{ceo}}}}}}}}}={\delta }_{2}-2{\delta }_{1}$$

The AlN thin films in this work were epitaxially grown on a *c*-plane sapphire substrate via metal-organic chemical vapour deposition^41,42^. As illustrated in Fig. 1b, the AlN epilayer exhibits a hexagonal wurtzite structure with a unit cell shown in the bottom, highlighting the non-centrosymmetry. We also show an overall 2-inch AlN-on-sapphire wafer featuring a broadband transparency and a favored film thickness (Fig. 1c)—both are crucial factors to ensure octave GVD control. Great attention was also paid to the film crystal quality and surface roughness for low-loss photonic applications. The AlN nanophotonic chips were manufactured following electron-beam lithography, chlorine-based dry etching, and silicon dioxide (SiO_2_) coating processes and were subsequently cleaved to expose waveguide facets^43^. The intrinsic optical quality factors (*Q*_int_) of the AlN resonators were characterized to be ~1–3 million depending on the waveguide geometries. The detailed film and device characterization is presented in “Methods” and Supplementary Fig. 1.

Since wurtzite AlN manifests optical anisotropy for vertically or horizontally-polarized light^44^, we engineer the waveguide structures for optimizing the performance of fundamental transverse magnetic (TM_00_) modes, which allows the harness of its largest *χ*^(2)^ susceptibility to ensure high-efficiency SHG. To expand microcomb spectra out of the anomalous GVD restriction, we exploit soliton-induced DW radiations by tailoring the resonator’s integrated dispersion (*D*_int_)^11^:

2 $${D}_{{{{{{{{\rm{int}}}}}}}}}=\frac{{D}_{2}}{2!}{\mu }^{2}+\frac{{D}_{3}}{3!}{\mu }^{3}+\mathop{\sum}\limits_{i\ge 4}\frac{{D}_{i}}{i!}{\mu }^{i}$$

where *D*_2_, *D*_3_, and *D*_*i*_ are *i*_th_-order GVD parameters, while *μ* indexes the relative azimuth mode number with respect to the pump (*μ* = 0). In the dispersion modeling, we have accounted for both the material (AlN) and geometry (cross section, bending radius, and slanted sidewall) dispersion^42^. To prevent avoided mode crossing from interrupting soliton mode-locking, we adopt a weak pulley-coupling configuration (concentric angle of 6°), which helps suppress the excitation of higher-order resonator modes (Supplementary Fig. 1c).

### Octave soliton microcombs

**Fig. 2:**
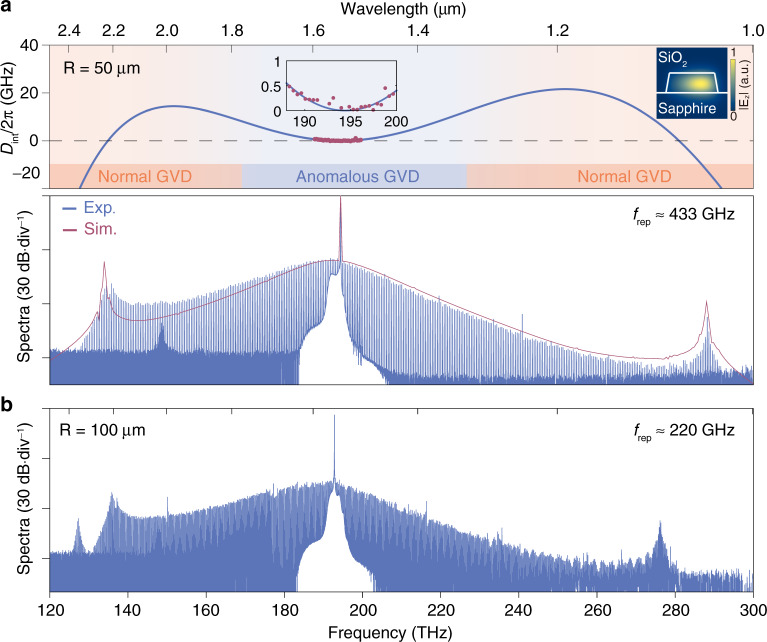
Octave soliton microcombs at hundreds of gigahertz repetition rates. **a** Top: integrated dispersion (*D*_int_) of a 50 μm-radius (R) AlN resonator (cross section: 1.0 × 2.3 μm^2^), where the anomalous and normal group velocity dispersion (GVD) regimes are indicated by solid light blue and orange colors in the bottom. Insets: zoom-in view of measured (red dots) and simulated (blue curve) *D*_int_ values in the center, and electric field abs(*E*_*z*_) of the TM_00_ resonator mode with a normalized color bar in the right. Bottom: soliton microcomb spectra from the experiment (Exp., blue curve) and simulation (Sim., red curve) at an on-chip pump power of ~390 mW. The resonator *Q*_int_ is 1.6 million and the repetition rate (*f*_rep_) is estimated to be around 433 GHz. **b** Soliton microcomb spectrum from a 100 μm-radius AlN resonator (cross section: 1.0 × 3.5 μm^2^) with a decreased *f*_rep_ of ~220 GHz. The applied pump power is ~1 W at a resonator *Q*_int_ of 2.5 million.

Our GVD engineered AlN resonators are coated with a SiO_2_ protection layer, making it less susceptible to the ambient compared with the air-cladded Si_3_N_4_ counterpart^25–29^. An example of the resonator modal profile is shown in the inset of Fig. 2a. The top panel of Fig. 2a plots the *D*_int_ curve from a 50 μm-radius AlN resonator through numerical simulation (see “Methods”). In spite of the limited anomalous GVD window (solid light blue region), octave microcomb operation is feasible via DW radiations at phase-matching conditions *D*_int_ = 0, allowing for spectral extension into normal GVD regimes (solid light orange regions). Note that the occurrence of such dual DWs benefits from the optimal film thickness in our AlN system, while the DW separation is agilely adjustable over one octave through the control of resonator’s dimensions (Supplementary Fig. 2). Around the telecom band, the *D*_int_ value (red dots) was characterized by calibrating the resonator’s transmission with a fiber-based Mach-Zehnder interferometer^15,17^. The experimental result matches well with the simulated one (inset of Fig. 2a) with an extracted *D*_2_/2*π* of ~6.12 MHz.

We then explore soliton mode-locking based on a rapid frequency scan scheme to address the abrupt intracavity thermal variation associated with transitions into soliton states^15^. The soliton spectrum is recorded using two grating-based optical spectrum analyzers (OSAs, coverage of 350–1750 nm and 1500–3400 nm). The experimental setup is detailed in Supplementary Fig. 4. The bottom panel of Fig. 2a plots the soliton spectrum from a 50 μm-radius AlN resonator, featuring a moderate *f*_rep_ of 433 GHz and an observable spectral span of 1.05–2.4 μm, exceeding one optical octave. Meanwhile, soliton-induced DW radiations occur at both ends of the spectrum, in agreement with the predicted *D*_int_ curves. Note that the high-frequency DW exhibits an evident blue shift from the *D*_int_ = 0 frequency, which is mainly ascribed to Raman-induced soliton red shifts (relative to the pump frequency)^45,46^. The soliton recoils make less impact here due to dual DW radiations^47^. This conclusion is supported by our modeling when comparing the soliton spectra with and without Raman effects (Supplementary Fig. 2b).

The single crystal nature of AlN thin films permits reproducible optical refractive indices in each manufacture run. This, in combination with their uniform film thickness control, leads to a high predictability for the dispersion engineering, making it feasible to predict octave soliton combs at various repetition rates. For instance, our GVD model indicates that octave spectra with repetition rates further decreased by two times are anticipated from 100 μm-radius AlN resonators at optimal widths of 3.3–3.5 μm (Supplementary Fig. 3a). Figure 2b plots the recorded soliton comb spectrum at a resonator width of 3.5 μm, where a *f*_rep_ of ~220 GHz and dual DWs separated by more than one octave are achieved simultaneously. Such a low *f*_rep_ is amenable for direct photodetection with state-of-the-art unitravelling-carrier photodiodes^48^. We also noticed the occurrence of a weak sharp spectrum around 130 THz, which might arise from modified local GVD due to avoided mode crossing^49^. In our nanophotonic platform, we could further predict resonator geometries for achieving octave solitons with an electronically detectable *f*_rep_ of ~109 GHz (Supplementary Fig. 3b). Nonetheless, the strong competition between Kerr nonlinearities and stimulated Raman scattering (SRS) must be taken into account since the free spectral range (FSR) of the resonator is already smaller than the ${A}_{1}^{{{{{{{{\rm{TO}}}}}}}}}$ phonon linewidth (~138 GHz) in AlN epilayers^42^.

**Fig. 3:**
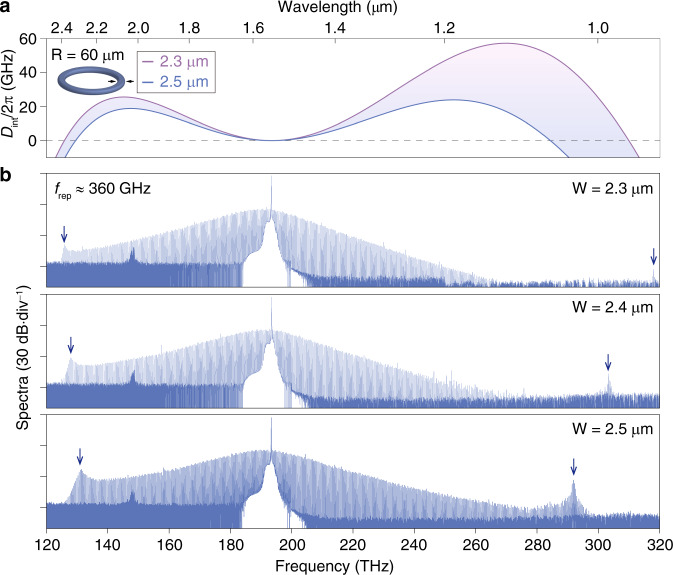
Octave soliton microcombs with agilely tunable spectra. **a** Engineered integrated dispersion (*D*_int_) curves of 60 μm-radius (R) AlN resonators at varied widths of 2.3–2.5 μm revealed by the colored shadow regime. **b** Corresponding soliton microcomb spectra at resonator widths of 2.3, 2.4, and 2.5 *μ*m from the top to bottom panel, respectively. The repetition rate (*f*_rep_) is ~360 GHz, while the vertical arrows in spectral wings indicate the emergence of DWs. Akin to Fig. 2a, high-frequency DWs here also exhibit an evident blue shift from the *D*_int_ = 0 position. From a sech^2^ fit, the corresponding temporal pulse duration is estimated to be ~23, 22, and 19 fs (from top to bottom), respectively.

Since the SHG from the auxiliary laser (1940–2000 nm) available in our laboratory is beyond the soliton spectral coverage shown in Fig. 2, we further adjust the resonator dimensions for extending microcomb spectra below 1 μm. As plotted in Fig. 3a, the phase-matching condition (*D*_int_ = 0) for high-frequency DW radiations below 1 μm is fulfilled by elevating the resonator radius to 60 μm while maintaining its width around 2.3 μm. In the meantime, low-frequency DWs could also be expected and their spectral separation is adjustable by controlling the resonator width. Guided by the tailored *D*_int_ curves, we fabricated the AlN resonators and recorded octave soliton spectra at a *f*_rep_ of ~360 GHz (Fig. 3b). Lithographic control of DW radiations (indicated by vertical arrows) is also verified by solely adjusting the resonator width, allowing the spectral extension below 1 μm (width of 2.3 or 2.4 μm). The low- and high-frequency DWs are found to exhibit distinct frequency shifting rates, consistent with the *D*_int_ prediction. The observable soliton spectra (from top to bottom of Fig. 3b) cover 1.5, 1.3, and 1.2 optical octaves by normalizing the total span (Δ*f*) to its beginning frequency (*f*_1_), that is Δ*f*/*f*_1_. Such a definition permits a fair comparison among soliton microcomb generation in distinct pump regimes across different material platforms, suggesting high competitiveness of our AlN microcomb span comparing to state-of-the-art values reported in Si_3_N_4_ microresonators^26^.

### On-chip second harmonic generator

We then explore the co-integration of SHG based on the *χ*^(2)^ susceptibility of AlN for matching the DW peak below 1 μm (middle panel of Fig. 3b). To fulfill the demanding requirement of spectral overlaps with the microcomb, we adopt a straight waveguide configuration, which allows a broader phase-matching condition albeit at the cost of reduced conversion efficiencies comparing to its counterpart using dual-resonant microresonators^34,50^. Through modeling, we predict an optimal waveguide width of ~1.38 μm for fulfilling the modal-phase-matching condition (Supplementary Fig. 5a), while the actual waveguide width was lithographically stepped from 1.32 to 1.46 μm (spacing of 5 nm) accounting for possible deviations during the manufacturing process.

**Fig. 4:**
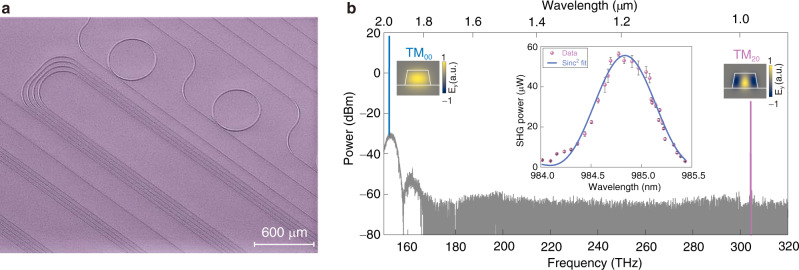
Second-harmonic generators. **a** Colored scanning electron microscope images of fabricated AlN nanophotonic chips composed of octave microcomb generators (microring resonators) and SHG waveguides (total length of 6 cm, not fully shown). The weak pulley-coupled geometry (concentric angle of 6°) in the resonators is not observable due to the large scale bar. **b** SHG spectra collected from a modal phase-matched waveguide (width of 1.395 μm) at an on-chip pump power of 355 mW. Insets: electric fields (*E*_*y*_, normalized color bars) of the pump (TM_00_) and SHG (TM_20_) modes, as well as wavelength-dependent SHG power (pink dots) with a sinc^2^-function fit (blue curve). The error bars reflect the SHG power variation from continuous three measurements, and the larger error in the center arises from the temperature fluctuation inside the waveguides, which in turn impacts the phase-matching condition^34^.

Figure 4 a shows a section of 6 cm-long SHG waveguides co-fabricated with the microcomb generator. At a fixed fundamental wavelength (1970 nm), we located the phase-matching waveguide at the width of 1.395 μm, close to the predicted width. The corresponding SHG spectra are plotted in Fig. 4b, where we achieve a high off-chip SHG power over 50 μW by boosting the fundamental pump power from a thulium-doped fiber amplifier to compensate the SHG efficiency (Supplementary Fig. 5b). In the meantime, the wavelength-dependent SHG power shown in the inset indicates a large 3-dB phase-matching bandwidth of ~0.8 nm, which, together with an external heater for thermal fine-tuning, is sufficient to cover the target comb lines for subsequent heterodyne beating.

### Nanophotonics-based *f*–2*f* interferometry

**Fig. 5:**
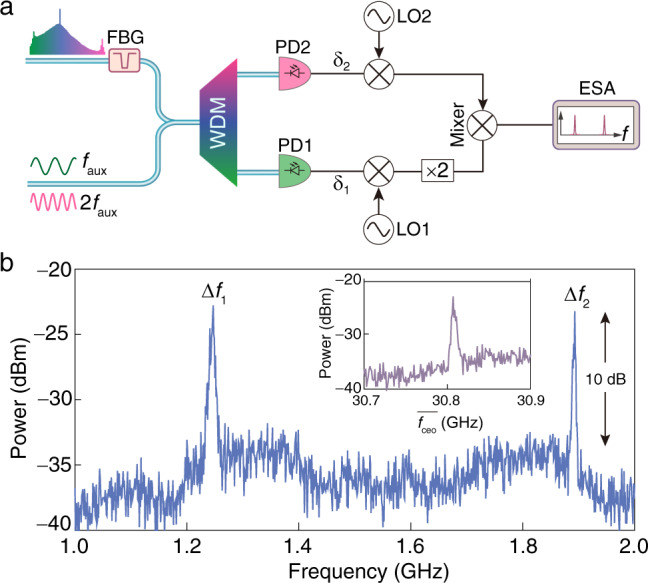
*f*–2*f* heterodyne measurement. **a** Schematic diagram for assessing the *f*_ceo_. The symbol “×2” indicates a radio frequency doubler. The experimental details are introduced in Supplementary Fig. 6c. **b** Free-running *f*–2*f* beatnotes after the down-conversion process, suggesting a signal-to-noise ratio of 10 dB at a resolution bandwidth of 1 MHz. The local oscillator frequencies *f*_LO1_ and *f*_LO2_ are chosen to be 11.8 and 9.1 GHz, respectively. Inset: the equivalent curve of $\overline{{f}_{{{{{{{{\rm{ceo}}}}}}}}}}$ = 2*f*_LO1_ + *f*_LO2_ – Δ*f*_2_.

By combining outgoing light from optimal AlN soliton and SHG generators on the calibrated OSAs, we are able to estimate the *f*–2*f* beatnote frequency to be approximately 32 GHz limited by the resolution of the OSAs. To electronically access the *f*_ceo_ signal in real time, we employ a scheme sketched in Fig. 5a. The recorded soliton spectrum after suppressing pump light by a fiber Bragg grating (FBG) indicates a high off-chip power close to −40 dBm for the high-frequency DW (Supplementary Fig. 6a). Meanwhile, a wavelength-division multiplexer (WDM) is utilized to separate the *f* and 2*f* frequency components before sent into the photodetectors (PDs). Two tunable radio frequency (RF) synthesizers are introduced as the local oscillators (LO1 and LO2) to down convert the photodetector signals for effective capture of the *f*–2*f* beat signal at a convenient low-frequency band with an electronic spectrum analyzer (ESA, range of 20 Hz–26.5 GHz).

As highlighted in Fig. 5b, we record two down-converted beatnotes of Δ*f*_1_ and Δ*f*_2_ with a signal-to-noise ratio (SNR) of 10 dB at a resolution bandwidth of 1 MHz. A much higher SNR is anticipated by applying a finer detection bandwidth upon locking the telecom pump laser as well as the *f*_ceo_ frequency^23,24^. The corresponding *f*–2*f* beatnote is $\overline{{f}_{{{{{{{{\rm{ceo}}}}}}}}}}$ = 2*f*_LO1_ + *f*_LO2_ – Δ*f*_2_ (inset of Fig. 5b) since the local oscillator frequencies *f*_LO1_ and *f*_LO2_ are chosen to be larger than beatnotes of *δ*_1_ and *δ*_2_. Based on the relative frequency positions of the auxiliary laser and its neighboring comb tooth (Supplementary Fig. 6b), we reach an actual *f*_ceo_ = FSR – $\overline{{f}_{{{{{{{{\rm{ceo}}}}}}}}}}$ due to the involvement of *f*_n_ and *f*_2n+1_ comb lines in the heterodyne beating. On the other hand, the *f*_LO1_ and *f*_LO2_ frequencies are freely adjustable up to 40 and 20 GHz in our scheme, which could further expand the accessible range of the *f*_ceo_ frequency based on the down-conversion process presented here. Meanwhile, the RF synthesizers are synchronized to a common external frequency reference, suggesting that the captured down-converted *f*–2*f* signals are available for further locking the comb teeth in a feedback loop^28^.

## Discussion

We demonstrate nanophotonics-based implementation of *f*–2*f* interferometry by leveraging *χ*^(3)^ octave solitons and *χ*^(2)^ SHG co-fabricated from a non-centrosymmetric AlN photonic platform. Thanks to agile GVD engineering offered by epitaxial AlN thin films, our octave soliton microcombs can be reliably produced with dual DWs and sub-THz repetition rates (220–433 GHz) that are accessible with unitravelling-carrier photodiodes. The overall soliton spectral span is adjustable up to 1.5 octaves, on a par with state-of-the-art values (1.4 octaves) reported in Si_3_N_4_ microresonators. We further perform the *f*_ceo_ measurement with the aid of an auxiliary laser for enabling SHG in phase-matched AlN waveguides, thus allowing for spectral overlap with the desired octave soliton.

For future development, the spectral restriction of octave solitons for matching with the auxiliary laser wavelength can be relaxed by exploiting high-efficiency SHG in dual-resonant microresonators, which allows direct doubling of a selected comb line in the low frequency DW band^51^. Meanwhile, the octave-spanning microcomb’s repetition rate can be further reduced by leveraging on-chip Pockels electro-optical frequency division^52^. By shifting the phase matching condition for SHG, it is also possible to extend octave solitons into the near-visible band, giving access to self-locked near-visible microcombs for precision metrology. Our results represent an important milestone to unlock the potentials of octave microcomb technologies for portable applications.

## Methods

### Nanofabrication

The surface roughness and crystal quality of our AlN epilayer were respectively characterized by an atomic force microscope and an X-ray diffraction scan, indicating a root-mean-square roughness of 0.2 nm in 1 × 1 *μ*m^2^ region and an FWHM linewidth of ~46 and 1000 arcsec along [002] and [102] crystal orientations, respectively. The film thickness was mapped by a spectroscopic ellipsometer (J.A. Woollam M-2000), providing a quick and preliminary selection of the desired AlN piece for octave soliton generation with dual DWs. As shown in Supplementary Fig. 1a, in spite of varied film thicknesses across a 2-inch AlN wafer, we can reliably locate the desired region for reproducible octave device fabrication.

To further reduce the propagation loss, the AlN photonic chips were annealed at 1000 °C for 2 h. The resonator *Q*-factors were probed by sweeping a tunable laser (Santec TSL-710) across the cavity resonances and then fitted by a Lorentzian function. In the 100 μm-radius AlN resonators (width of 3.5 μm), we achieve a recorded *Q*_int_ of 3.0 million, while the 50 μm-radius resonators (width of 2.3 μm) exhibit a decreased *Q*_int_ of 1.6 million, indicating the dominant sidewall scattering loss of our current fabrication technology. The related resonance curves are plotted in Supplementary Fig. 1c and d.

### Numerical simulation

The *D*_int_ of the AlN resonators is investigated using a finite element method (FEM) by simultaneously accounting for the material and geometric chromatic dispersion. The overall *D*_int_ value is approximated with a fifth-order polynomial fit applied to the simulated modal angular frequencies: *ω*_*μ*_ = *ω*_0_ + *μD*_1_ + *D*_int_, where *D*_1_/(2*π*) is the resonator’s FSR at the pump mode *μ* = 0.

The spectral dynamics of octave soliton microcombs is numerically explored based on nonlinear coupled mode equations by incorporating the Raman effect^46,53^:

3 $$\frac{\partial }{\partial t}{a}_{\mu }=-\Big(\frac{{\kappa }_{\mu }}{2}+i{{{\Delta }}}_{\mu }^{a}\Big){a}_{\mu }+i{g}_{{{{{{{{\rm{K}}}}}}}}}\mathop{\sum}\limits_{k,l,n}{a}_{k}^{* }{a}_{l}{a}_{n}\delta (l+n-k-\mu )\\ -\,i{g}_{{{{{{{{\rm{R}}}}}}}}}\mathop{\sum}\limits_{k,l}{a}_{l}\left[\right.{{{{{{{{\mathcal{R}}}}}}}}}_{k}\delta (l+k-\mu )+{{{{{{{{\mathcal{R}}}}}}}}}_{k}^{* }\delta (l-k-\mu )\left]\right.+{\xi }_{{{{{{{{\rm{P}}}}}}}}}$$

4 $$\frac{\partial }{\partial t}{{{{{{{{\mathcal{R}}}}}}}}}_{\mu }=-\Big(\frac{{\gamma }_{{{{{{{{\rm{R}}}}}}}}}}{2}+i{{{\Delta }}}_{\mu }^{{{{{{{{\rm{R}}}}}}}}}\Big){{{{{{{{\mathcal{R}}}}}}}}}_{\mu }-i{g}_{{{{{{{{\rm{R}}}}}}}}}\mathop{\sum}\limits_{k,l}{a}_{k}^{* }{a}_{l}\delta (l-k-\mu )$$

Here *a* and ${{{{{{{\mathcal{R}}}}}}}}$ are the mode amplitudes of cavity photons and Raman phonons with subscripts *k*, *l*, *n* being the mode indices, while *g*_K_ and *g*_R_ represent the nonlinear coupling strength of Kerr and Raman processes, respectively. The driving signal strength is ${\xi }_{{{{{{{{\rm{P}}}}}}}}}=\delta (\mu )\sqrt{\frac{{\kappa }_{{{{{{{{\rm{e}}}}}}}},0}{P}_{{{{{{{{\rm{in}}}}}}}}}}{\hslash {\omega }_{{{{{{{{\rm{p}}}}}}}}}}}$ at an on-chip pump power *P*_in_, *κ*_*μ*_ (*κ*_e,*μ*_) denotes the total (external) cavity decay rate of the *μ*^th^ photon mode, and *γ*_R_ is the Raman phonon decay rate. The detuning from a *D*_1_-spaced frequency grid is indicated by ${{{\Delta }}}_{\mu }^{a}$ = *ω*_*μ*_ – *ω*_P_ – *μD*_1_ and ${{{\Delta }}}_{\mu }^{{{{{{{{\rm{R}}}}}}}}}$ = *ω*_R_ – *μD*_1_ with *ω*_P_ and *ω*_R_ being pump and Raman shift angular frequencies, respectively.

In the simulation, we set the time derivative of Raman items in Eq. (4) to zero to speed up the computation since the decay rate of phonons is much larger than that of photons. We also consider frequency-independent *κ*_*μ*_/(2*π*) ≈ 120 MHz and *κ*_e,*μ*_/(2*π*) ≈ 75 MHz based on measured *Q*-factors of 50 μm-radius AlN resonators (Supplementary Fig. 1c). Because incident light is TM-polarized, the involved ${A}_{1}^{{{{{{{{\rm{TO}}}}}}}}}$ Raman phonon in AlN exhibits an *ω*_R_/(2*π*) ≈ 18.3 THz with an FWHM of *γ*_R_/(2*π*) ≈ 138 GHz^41^. The *g*_K_/2*π* is calculated to be 0.73 Hz for a given nonlinear refractive index *n*_2_ = 2.3 × 10^−19^ m^2^/W, while an optimal *g*_R_/2*π* = 0.29 MHz is adopted, resulting in a soliton spectrum matching well with the measured one in Fig. 2a. The simulated high frequency DW also exhibits an evident blue shift comparing with the case of *g*_R_/2*π* = 0 MHz (Supplementary Fig. 2b).

## Supplementary information

Supplementary Information

## Data Availability

The source data that support the findings of this study are available in figshare repository: 10.6084/m9.figshare.15167895.v1.

## References

[1] Fortier T, Baumann E (2019). 20 years of developments in optical frequency comb technology and applications. Commun. Phys..

[2] Waldburger D (2019). Tightly locked optical frequency comb from a semiconductor disk laser. Opt. Express.

[3] Zhang M (2019). Broadband electro-optic frequency comb generation in a lithium niobate microring resonator. Nature.

[4] Gaeta AL, Lipson M, Kippenberg TJ (2019). Photonic-chip-based frequency combs. Nat. Photon..

[5] Pasquazi A (2018). Micro-combs: a novel generation of optical sources. Phys. Rep..

[6] Kippenberg TJ, Gaeta AL, Lipson M, Gorodetsky ML (2018). Dissipative kerr solitons in optical microresonators. Science.

[7] Stern B, Ji X, Okawachi Y, Gaeta AL, Lipson M (2018). Battery-operated integrated frequency comb generator. Nature.

[8] Raja AS (2019). Electrically pumped photonic integrated soliton microcomb. Nat. Commun..

[9] Herr T (2014). Temporal solitons in optical microresonators. Nat. Photon..

[10] Xue X (2015). Mode-locked dark pulse kerr combs in normal-dispersion microresonators. Nat. Photon..

[11] Brasch V (2015). Photonic chip-based optical frequency comb using soliton cherenkov radiation. Science.

[12] Yi X, Yang Q-F, Yang KY, Suh M-G, Vahala K (2015). Soliton frequency comb at microwave rates in a high-q silica microresonator. Optica.

[13] Joshi C (2016). Thermally controlled comb generation and soliton modelocking in microresonators. Opt. Lett..

[14] Yu M, Okawachi Y, Griffith AG, Lipson M, Gaeta AL (2016). Mode-locked mid-infrared frequency combs in a silicon microresonator. Optica.

[15] Gong Z (2018). High-fidelity cavity soliton generation in crystalline AlN micro-ring resonators. Opt. Lett..

[16] He Y (2019). Self-starting bi-chromatic linbo3 soliton microcomb. Optica.

[17] Gong Z (2019). Soliton microcomb generation at 2 μm in z-cut lithium niobate microring resonators. Opt. Lett..

[18] Yang Q-F, Yi X, Yang KY, Vahala K (2016). Stokes solitons in optical microcavities. Nat. Phys..

[19] Guo H (2016). Universal dynamics and deterministic switching of dissipative kerr solitons in optical microresonators. Nat. Phys..

[20] Cole DC, Lamb ES, Del’Haye P, Diddams SA, Papp SB (2017). Soliton crystals in kerr resonators. Nat. Photon..

[21] Jones DJ (2000). Carrier-envelope phase control of femtosecond mode-locked lasers and direct optical frequency synthesis. Science.

[22] Giunta M (2019). 20 years and 20 decimal digits: a journey with optical frequency combs. IEEE Photon. Technol. Lett..

[23] Spencer DT (2018). An optical-frequency synthesizer using integrated photonics. Nature.

[24] Newman ZL (2019). Architecture for the photonic integration of an optical atomic clock. Optica.

[25] Li Q (2017). Stably accessing octave-spanning microresonator frequency combs in the soliton regime. Optica.

[26] Pfeiffer MHP (2017). Octave-spanning dissipative kerr soliton frequency combs in si_3_n_4_ microresonators. Optica.

[27] Yu S-P (2019). Tuning kerr-soliton frequency combs to atomic resonances. Phys. Rev. Appl..

[28] Drake TE (2019). Terahertz-rate kerr-microresonator optical clockwork. Phys. Rev. X.

[29] Briles TC (2018). Interlocking kerr-microresonator frequency combs for microwave to optical synthesis. Opt. Lett..

[30] Xiong C (2012). Aluminum nitride as a new material for chip-scale optomechanics and nonlinear optics. New J. Phys..

[31] Kneissl M, Seong T-Y, Han J, Amano H (2019). The emergence and prospects of deep-ultraviolet light-emitting diode technologies. Nat. Photon..

[32] Bishop SG (2020). Room temperature quantum emitter in aluminum nitride. ACS Photonics.

[33] Lu T-J (2020). Bright high-purity quantum emitters in aluminum nitride integrated photonics. ACS Photonics.

[34] Bruch AW (2018). 17000%/w second-harmonic conversion efficiency in single-crystalline aluminum nitride microresonators. Appl. Phys. Lett..

[35] Liu X (2019). Beyond 100 THz-spanning ultraviolet frequency combs in a non-centrosymmetric crystalline waveguide. Nat. Commun..

[36] Bruch AW (2021). Pockels soliton microcomb. Nat. Photon..

[37] Chang L (2018). Heterogeneously integrated gaas waveguides on insulator for efficient frequency conversion. Laser & Photon. Rev..

[38] Hickstein DD (2017). Ultrabroadband supercontinuum generation and frequency-comb stabilization using on-chip waveguides with both cubic and quadratic nonlinearities. Phys. Rev. Appl..

[39] Yu M, Desiatov B, Okawachi Y, Gaeta AL, Lončar M (2019). Coherent two-octave-spanning supercontinuum generation in lithium-niobate waveguides. Opt. Lett..

[40] Okawachi Y (2020). Chip-based self-referencing using integrated lithium niobate waveguides. Optica.

[41] Liu X (2017). Integrated continuous-wave aluminum nitride raman laser. Optica.

[42] Liu X (2018). Integrated high-q crystalline aln microresonators for broadband kerr and raman frequency combs. ACS Photonics.

[43] Liu X (2018). Ultra-high-q uv microring resonators based on a single-crystalline aln platform. Optica.

[44] Majkić A (2017). Optical nonlinear and electro-optical coefficients in bulk aluminium nitride single crystals. Phys. Status Solidi B.

[45] Karpov M (2016). Raman self-frequency shift of dissipative kerr solitons in an optical microresonator. Phys. Rev. Lett..

[46] Gong Z, Liu X, Xu Y, Tang HX (2020). Near-octave lithium niobate soliton microcomb. Optica.

[47] Cherenkov A, Lobanov V, Gorodetsky M (2017). Dissipative kerr solitons and cherenkov radiation in optical microresonators with third-order dispersion. Phys. Rev. A.

[48] Zhang S (2019). Terahertz wave generation using a soliton microcomb. Opt. Express.

[49] Yi X (2017). Single-mode dispersive waves and soliton microcomb dynamics. Nat. Commun..

[50] Bruch AW, Liu X, Surya JB, Zou C-L, Tang HX (2019). On-chip *χ*(2) microring optical parametric oscillator. Optica.

[51] Surya JB, Guo X, Zou C-L, Tang HX (2018). Control of second-harmonic generation in doubly resonant aluminum nitride microrings to address a rubidium two-photon clock transition. Opt. Lett..

[52] Li J, Yi X, Lee H, Diddams SA, Vahala KJ (2014). Electro-optical frequency division and stable microwave synthesis. Science.

[53] Gong Z (2020). Photonic dissipation control for kerr soliton generation in strongly raman-active media. Phys. Rev. Lett..

