# Supplementary material for: Aluminum nitride nanophotonics for beyond-octave soliton microcomb generation and self-referencing
Source: Nat Commun. 2021 Sep 14;12:5428. doi: 10.1038/s41467-021-25751-9 (PMC8440561; doi:10.1038/s41467-021-25751-9)
Supplement: Supplementary file 1 — Supplementary Information [file 41467_2021_25751_MOESM1_ESM.pdf]

Supplementary Information:

Aluminum nitride nanophotonics for beyond-octave soliton microcomb generation and self-referencing

Xianwen Liu,[1,2] Zheng Gong,[1] Alexander W. Bruch,[1] Joshua B. Surya,[1] Juanjuan Lu,[1] and Hong X. Tang[1,3]

[1] *Department of Electrical Engineering, Yale University, New Haven, CT 06511, USA*

[2] *Current address: School of Optics and Photonics, Beijing Institute of Technology, Beijing, China*

[3] *Corresponding author: hong.tang@yale.edu*

I. DEVICE FABRICATION

All devices are patterned from 2-inch crystalline AlN-on-sapphire wafers with 1000 nm-thick AlN epilayers. To ensure robust dispersion engineering for reproducible octave-soliton generation, we first characterized the uniformity of film thickness across the wafer. As shown in Supplementary Fig. 1a, the film thickness of the wafer is very close to target growth thickness apart from the edge of the wafer (colored by purple). By intentionally choosing the desired thickness region, octave-soliton generation can be reliably realized using our current nanofabrication technology. For low-loss nanophotonic applications, we also paid attention to the crystal quality of the AlN thin film, whose root-mean-square surface roughness was characterized to be as low as 0.2 nm in a $1 \times 1\,\mu\mathrm{m}^2$ region. The result is presented in Supplementary Fig. 1b.

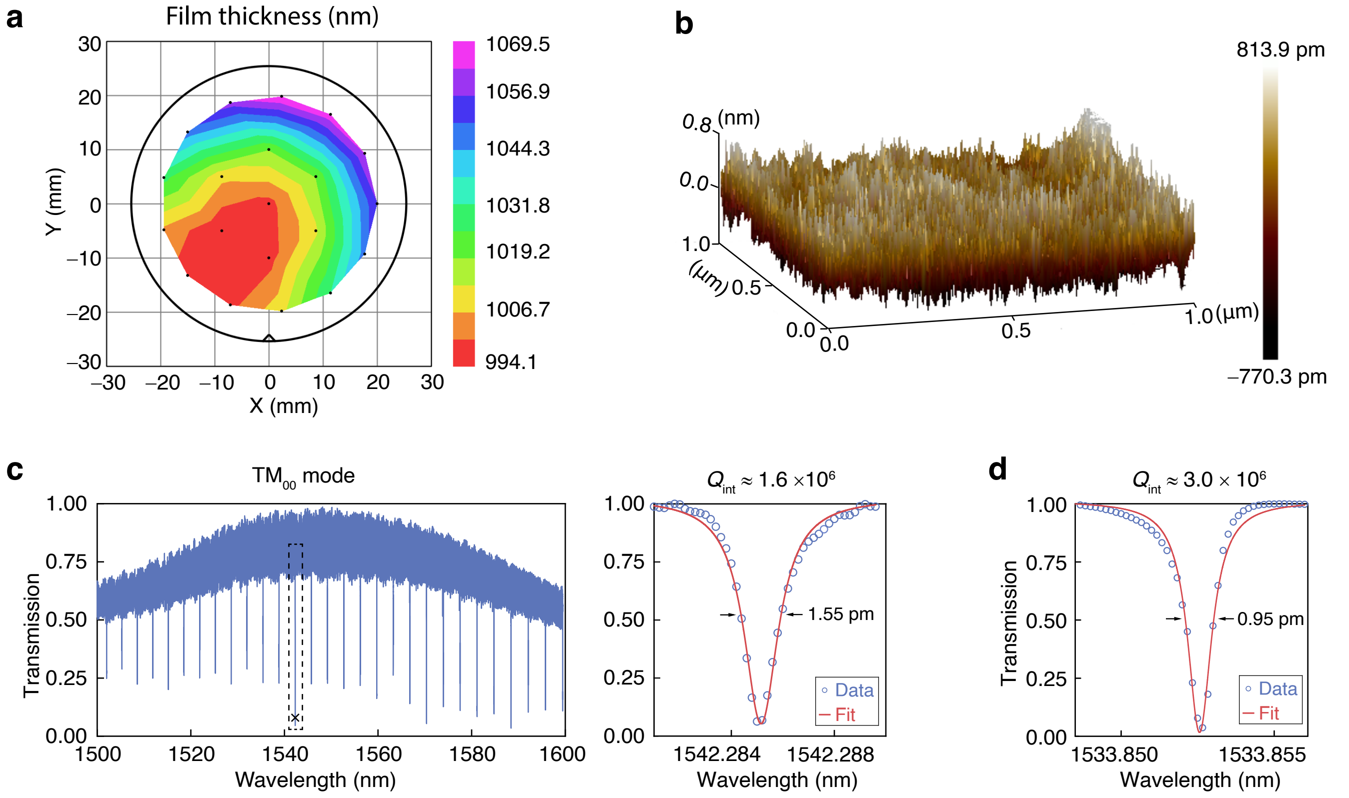

Supplementary Figure 1. Nanophotonic device fabrication. a. Wafer-scale thickness mapping of a 2-inch AlN wafer using a spectroscopic ellipsometer. The right color bar indicates the corresponding thickness variation. **b.** Surface roughness characterization of the AlN film using an atomic force microscope. **c.** Transmittance of a $50\,\mu$m-radius AlN resonator (width = $2.3\,\mu$m) for octave-soliton generation. A zoom-in view of the resonance (indicated by dashed lines) around 1542 nm is shown in the right side, revealing a Q_{int} of ∼1.6 million. **d.** Resonance from an $100\,\mu$m-radius AlN resonator (width = $3.5\,\mu$m), highlighting a reduced resonant linewidth and an improved Q_{int} of ∼3.0 million.

An example of the resonator transmittance is shown in Supplementary Fig. 1c, where the fundamental transverse magnetic (TM_{00}) mode is effectively excited while higher-order modes are suppressed by adopting a weak pulley waveguide coupling configuration (concentric angle of $6°$) according to our experiment. The intrinsic quality-factors (Q_{int}) are then extracted from a Lorentz

fit of the resonance curve at under-coupled conditions. The AlN resonators engineered for octave-soliton generation exhibits a dimension-dependent Q_{int} of 1.6 million and 3.0 million for the devices with radii of 50 μm (width = 2.3 μm, right of Supplementary Fig. 1c) and 100 μm (width = 3.5 μm, Supplementary Fig. 1d), suggesting the dominant sidewall scattering loss. Further improvement of the Q-factors can be envisioned by leveraging the racetrack resonators, where the straight portions exhibit a much smoother sidewall [1].

II. OCTAVE SOLITON ENGINEERING & CHARACTERIZATION

We note that the AlN resonator's integrated dispersion (D_{int}) is highly susceptible to the film thickness variation, which affects octave soliton generation with phase-matched dual dispersive waves (DWs). As plotted in Supplementary Fig. 2a, when the resonator height deviates from an optimal value of 1.00 μm (blue curve), such as increasing to 1.05 μm or decreasing to 0.95 μm, a larger dispersion barrier or a narrower dispersion window will occur on both sides of the D_{int} curve, preventing from octave spectral extension via DW radiations. As a result, we intentionally locate the AlN piece with the desired thickness around 1000 nm (Supplementary Fig. 1a) for the octave-soliton device fabrication.

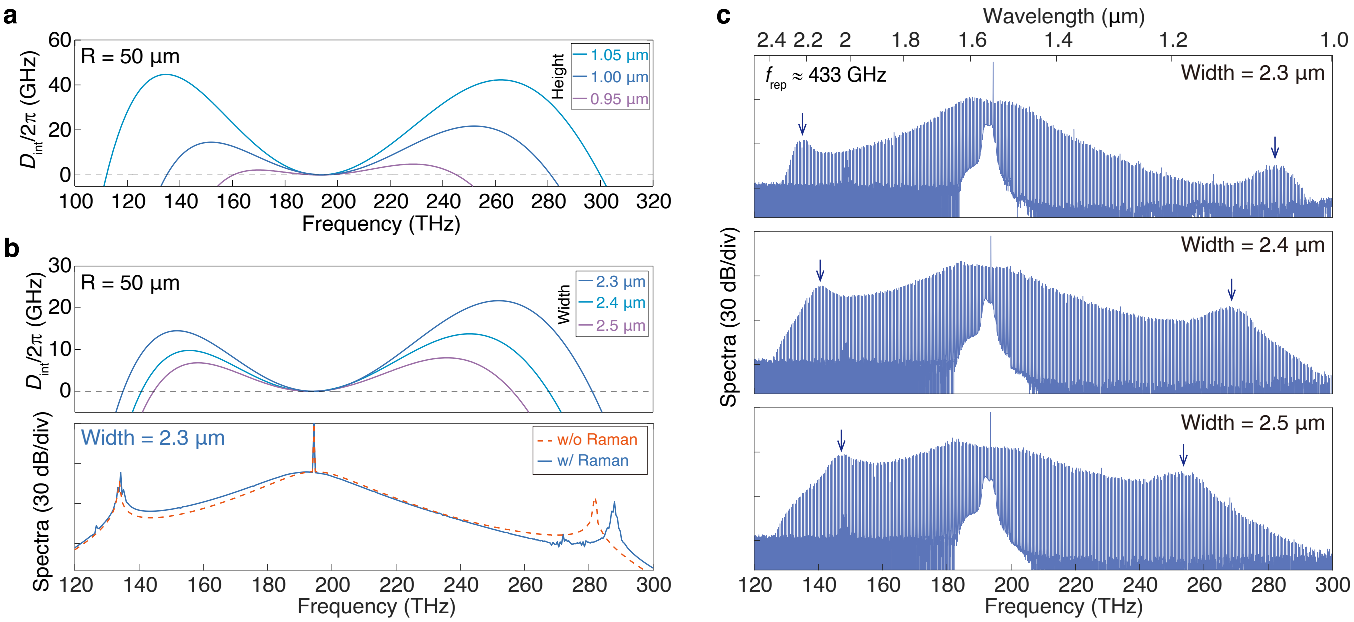

Supplementary Figure 2. Dispersion engineering for octave soliton generation. **a**. Height-dependent integrated dispersion (D_{int}) of 50 μm-radius AlN resonators at a fixed width of 2.3 μm. **b**. Top: width-dependent D_{int} curves (radius of 50 μm, height of 1.00 μm). Bottom: numerically simulated soliton comb spectra without (orange) or with (blue) the influence of Raman effects for the D_{int} curve at a resonator width of 2.3 μm (height of 1.0 μm). **c**. Experimentally recorded chaotic comb spectra at a varied resonator width of 2.3, 2.4 and 2.5 μm. The vertical arrows indicate the emergence of dispersive wave-like envelopes, and the corresponding D_{int} curves are shown in the top panel of **b**.

At an optimal height of 1.0 μm, the D_{int} curve can be further engineered by tailoring the resonator width. The result is shown in the top panel of Supplementary Fig. 2b, where the phase-matching

condition ($D_{\mathrm{int}}=0$) for DW radiations is readily adjusted beyond one octave span when reducing the resonator width from 2.5 to 2.3 μm. We then numerically investigate the octave soliton spectrum for the D_{int} curve at a width of 2.3 μm. As shown in the bottom panel of Supplementary Fig. 2b, the high-frequency DW exhibits an evident blue shift from the $D_{\mathrm{int}}=0$ position when accounting for the Raman effect, which matches wells with our experimental result in Fig. 2a of the main text. The underlying mechanism for this spectral shift is attributed to the Raman-induced soliton red shift in the spectral center, which in turn blue shifts the high-frequency DW [2]. Supplementary Fig. 2c plots the noise-state comb spectra recorded from the dispersion engineered AlN resonators (radius of 50 μm, width of 2.3–2.5 μm). It is found that the DW-like envelops at both ends of the spectra exhibit an evident shift when varying the resonator width, in good agreement with the D_{int} curve prediction (top panel of Supplementary Fig. 2b). In our experiment, the corresponding soliton spectrum can be captured at a resonator width of 2.3 μm (see Fig. 2a in the main text), while it is inaccessible at the width of 2.4 and 2.5 μm due to the occurrence of Raman lines in the intermediate state, thus hampering soliton mode-locking.

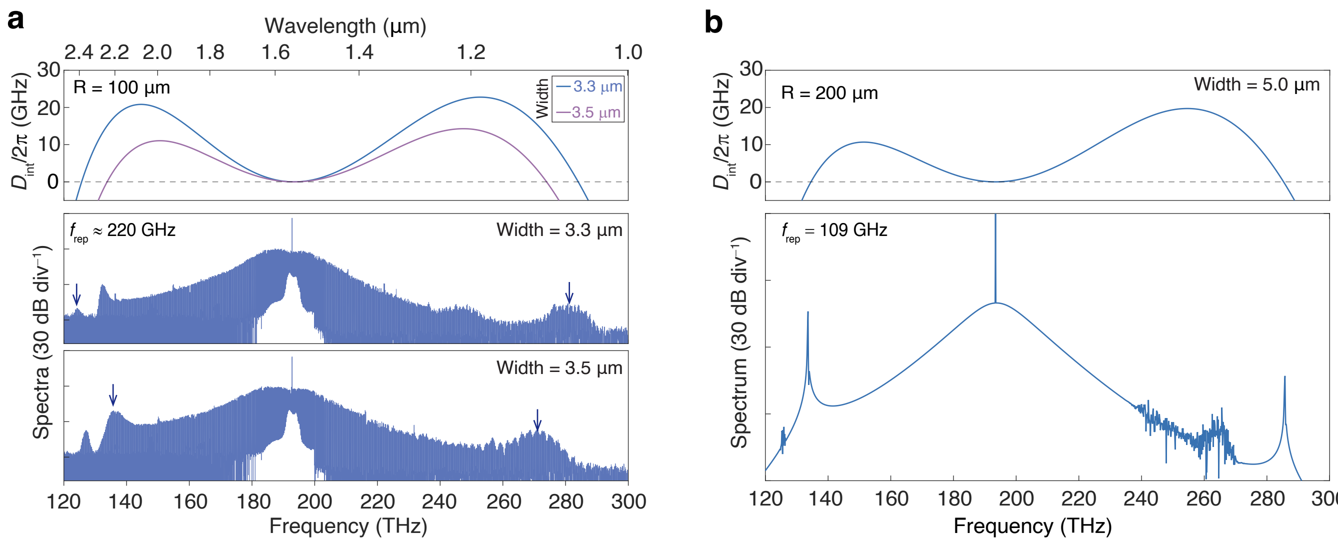

Supplementary Figure 3. Dispersion engineering for repetition rate-detectable octave solitons. a. Top: D_{int} curves of engineered AlN resonators at a radius of 100 μm and varied widths of 3.3 and 3.5 μm. Bottom: captured noise-state comb spectra with a reduced repetition rate of ∼220 GHz. The vertical arrows indicate the emergence of DW-like peaks. **b.** Top: D_{int} curve of AlN resonators at an increasing radius of 200 μm and an optimal width of 5.0 μm. Bottom: simulated soliton spectrum at an on-chip pump power of 100 mW, highlighting a reduced repetition rate of 109 GHz.

By further engineering the resonator dimensions, we are able to achieve octave solitons with repetition rates reduced by two times. As shown in the top panel of Supplementary Fig. 3a, at an elevated resonator radius of 100 μm, the phase-matching conditions for separated dual DWs by one optical octave are accessible from engineered D_{int} curves at optimal resonator widths of 3.3 and 3.5 μm. This prediction is verified by the recorded comb spectra with repetition rates (f_{rep}) of ∼220 GHz (bottom panel of Supplementary Fig. 3a), where dual DW-like envelopes indicated by vertical arrows match well with the $D_{\mathrm{int}}=0$ condition in each case. The abnormal spectral

peaks observed in the low-frequency region might arise from the avoided mode-crossing due to imperfections in the device fabrication [3], which is not included in our dispersion modeling. The soliton comb spectrum was captured at a resonator width of 3.5 μm (see Fig. 2b of the main text), while the occurrence of intermediate Raman lines prevents from soliton mode-locking for the case of a resonator width = 3.3 μm.

Note that the agile dispersion engineering in our material system also offers the capability to achieve octave solitons with electronically detectable repetition rates by commercial high-speed photodetectors (bandwidth > 100 GHz). As shown in Supplementary Fig. 3b, upon increasing the resonator radius to 200 μm, it is possible to achieve a low free spectral range (FSR) of 109 GHz and an optimal $D_{\rm int}$ curve (top panel) for octave soliton generation at a optimal resonator width of 5.0 μm. The corresponding soliton spectrum was numerically investigated and presented in the bottom panel. Here we choose an aspirational $Q_{\rm int}$ of 10 million at critical-coupled conditions for enabling octave soliton generation at a low on-chip pump power of 100 mW. Since the resonator's FSR is already smaller than the $A_1^{\rm TO}$ phonon linewidth ($\sim$138 GHz) of crystal AlN films [4], the intracavity stimulated Raman scattering will be significant, thereby prevents from soliton mode-locking. This issue can be addressed by introducing a large coupling loss at the Stokes wavelengths, which reduces the effective Raman gain [2]. For the numerical investigation here, we ignore the influence of Raman effects.

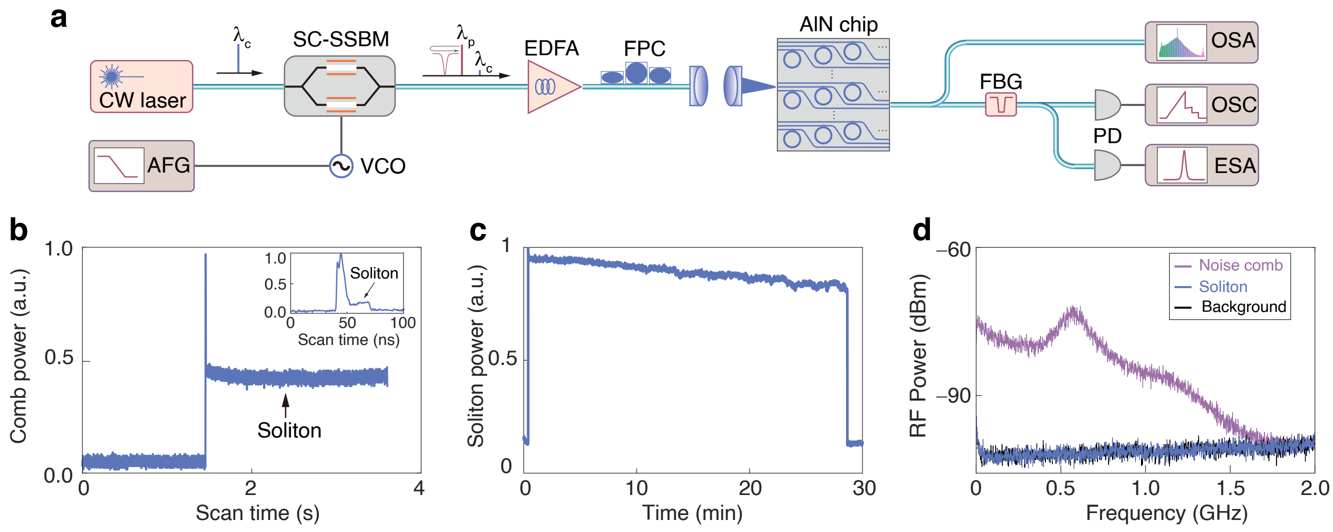

Supplementary Figure 4. Octave-soliton characterization. **a**. Sketch of the experimental apparatus. The SC-SSBM produces a blue-shifted sideband (λ_p) relative to incident light (λ_c) from a continuous-wave (CW) laser. The sideband is then boosted by an erbium-doped fiber amplifier (EDFA) and aligned to the vertical polarization before entering the AlN chip via an aspherical lens pair. **b**. Comb power trace recorded in the OSC, indicating elongated soliton duration time beyond 2 s. Inset: initial soliton lifetime of $\sim$20 ns. **c**. Free-running soliton power trace as a function of the time. **d**. Radio-frequency (RF) beating properties of chaotic (red) and soliton microcombs (blue) comparing with the PD background (black).

The experimental setup for our octave soliton generation is sketched in Supplementary Fig. 4a. The transition from chaotic to soliton states typically accompanies a notable intracavity power

drop, which in turn renders thermo cooling of the resonator with blue-shifted resonances, hindering stable soliton formation [5]. We address this obstacle using rapid frequency scan schemes based on a suppressed-carrier single sideband modulator (SC-SSBM) [6]. The SC-SSBM is driven by a voltage controller oscillator (VCO) connected to an arbitrary function generator (AFG), allowing rapid frequency shifting (up to 500 MHz/ns) across the resonance at a timescale far beyond the thermal-optic response (microseconds).

For the characterization, light exiting the chip is collected by a bare fiber (mode diameter of 4 μm) before sent into two grating-based optical spectrum analyzers (OSAs, the other one is not shown) and two photodetectors (PDs) following by an oscilloscope (OSC) and an electronic spectrum analyzer (ESA). A fiber-Bragg grating (FBG) is also employed to suppress strong pump light. Supplementary Fig. 4b plots a typical comb power trace when entering the soliton state. In spite of the initial soliton lifetime of ~20 ns, we can elongate it beyond 2 s using the rapid frequency scan scheme. The corresponding octave-soliton spectrum is shown in Fig. 2a of the main text. Upon entering the soliton state, our octave comb maintains a high stability during the full experiment span until the fiber-to-chip coupling becomes misaligned as indicated in Supplementary Fig. 4c. We also evaluate the coherence of the spectrum by sending a portion of comb lines (after suppressing pump light) into a PD. As shown in Supplementary Fig. 4d, there is no evidence of low-frequency radio-frequency (RF) noise within a span of 2 GHz for the octave soliton comparing with the chaotic state, suggesting a high degree of coherence.

III. ON-CHIP SECOND HARMONIC GENERATION AND SELF-REFERENCING

To access the carrier-envelop offset frequency (f_{ceo}) of octave soliton combs, a set of AlN waveguides were co-fabricated for efficient second-harmonic generation (SHG) as described in Fig. 4a of the main text. Here we consider the modal-phase-matching case for the pump (TM_{00}) and SHG (TM_{20}) modes, for which we can obtain an optimal waveguide width around 1.38 μm to fulfill the phase-matching condition (inset of Supplementary Fig. 5a). By lithographically scanning the waveguide width at a spacing of 5 nm, we locate the phase-matching waveguide for producing SHG spectra shown in Fig. 4b of the main text. The insertion loss of the TM_{00} mode at 1970 nm was measured to be ~7 dB/facet, while the insertion loss of the TM_{20} mode at 985 nm is estimated to be around 15 dB/facet because of the small modal overlap between the high-order TM_{20} mode with the fiber mode. The calibrated on-chip SHG power (P_{SHG}) versus the pump power (P_{p}) is plotted in Supplementary Fig. 5a, where an on-chip SHG conversion efficiency $\eta_{\text{SHG}} = P_{\text{SHG}}/P_{\text{p}}^2$ is derived to be 0.012 W^{-1}.

We then analytically investigate the SHG efficiency with the coupled wave equation at a slowly varying amplitude approximation. Since the SHG mode (TM_{20}) is more susceptible to the waveguide sidewall roughness, we also include its propagation loss (α) into the model shown below (Supplementary Ref. [7]):

$$\frac{db}{dz} = -\frac{\alpha}{2}b + \frac{i{\omega_b}^2\chi^{(2)}\Gamma}{4k_b c^2}a^2\exp(i\Delta k z) \tag{1}$$

Here a and b are the slowly varying field amplitude of TM_{00} and TM_{20} waves along the waveguide direction z, while $k_q = n_b\omega_q/c$ is the propagation constant with n, ω, and c being the effective refractive index, angular frequency, and light speed in vacuum, respectively. The subscript q denotes the parameters of the mode a or b. Meanwhile, $\chi^{(2)}$ is the quadratic optical nonlinearity, Δk equals to $2k_a - k_b$, and Γ describes the modal overlap of a and b modes given by:

$$\Gamma = \frac{\int u_a^2 u_b^* dxdy}{(\int |u_a|^2 dxdy)(\int |u_b|^2 dxdy)^{1/2}} \tag{2}$$

where u_a and u_b indicate the transverse field distribution.

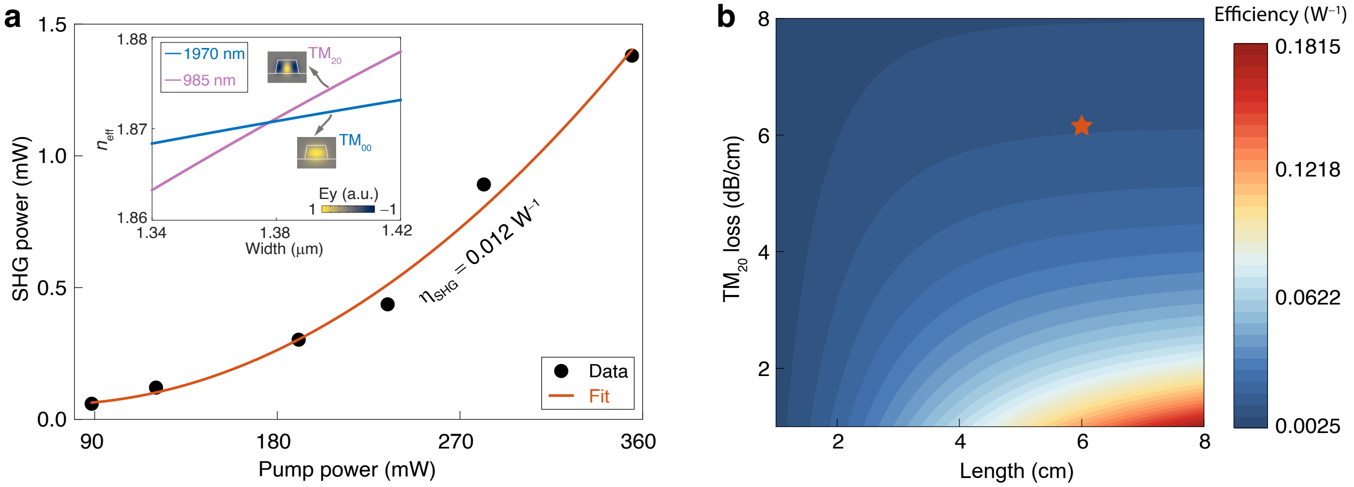

Supplementary Figure 5. SHG conversion efficiency. a. On-chip SHG power versus the pump power as well as the applied second-order polynomial fit (orange solid line). Inset: calculated effective refractive indices (n_{eff}) of TM_{00} (wavelength of 1970 nm) and second-order TM_{20} (wavelength of 985 nm) modes versus the waveguide width. **b**. Calculated SHG efficiency (indicated by the right color bar) as a function of the TM_{20} mode propagation loss and the waveguide length. The "red star" symbol corresponds to our experimentally estimated SHG efficiency.

By solving Supplementary Eq. (1) for deriving the SHG power $P_{\text{SHG}} = \frac{n_b\varepsilon_0 c|b|^2}{2}\int |u_b|^2 dxdy$, the on-chip SHG efficiency reads:

$$\eta_{\text{SHG}} = \frac{(\omega_a\chi^{(2)}\Gamma L)^2}{2n_a^2 n_b\varepsilon_0 c^3}\frac{\sinh^2(\alpha L/4) + \sin^2(\Delta kL/2)}{(\alpha L/4)^2 + (\Delta kL/2)^2} \tag{3}$$

Here ε_0 is the permittivity in vacuum and L is the overall waveguide length. Based on Supplementary Eq. (3), we calculate η_{SHG} at the phase-matching condition (i.e., $\Delta k = 0$) as shown in Supplementary Fig. 5b. It is evident that the mode propagation loss makes a singficant impact on the SHG efficiency in such a long waveguide. In the experiment, we adopt a waveguide length of 6 cm and the calculated η_{SHG} is found to agree with the experimental value when the TM_{20} loss is around 6 dB/cm. The result is in reasonable agreement with our experimentally extracted TM_{20} loss ($\sim$2 dB/cm at 780 nm [8]) when accounting for possible deviation from perfect phase-matching conditions in practical devices.

Based on the optimized octave soliton comb and SHG generator, we establish a f–$2f$ interferometer for accessing the f_{ceo} frequency. To enable efficient optical-to-electrical conversion in the PDs, the

residual pump light is suppressed by a broadband FBG and the recorded octave comb spectrum is presented in Supplementary Fig. 6a. By adjusting the auxiliary laser frequency ($f_{\rm aux}$) to overlap with one of the comb line, we produce a strong $2f_{\rm aux}$ tone in the proximity of $f_{\rm 2n}$ comb line. In our case, $2f_{\rm aux}$ is actually closer to the $f_{\rm 2n+1}$ comb line, which is then selected for implementing the f–$2f$ interferometry. These optical frequencies are monitored by two OSAs (350–1750 nm and 1500–3400 nm) at a resolution of 0.05 and 0.1 nm, respectively. The positions of relevant laser lines are sketched in Supplementary Fig. 6b, where a positive $f_{\rm ceo}$ frequency is ensured at the assigned comb line indices. By setting $\delta_1 = f_{\rm aux} - f_{\rm n}$ and $\delta_2 = f_{\rm 2n+1} - 2f_{\rm aux}$, we have $f_{\rm ceo} = 2f_{\rm n} - f_{\rm 2n} = {\rm FSR} - (\delta_2 + 2\delta_1)$.

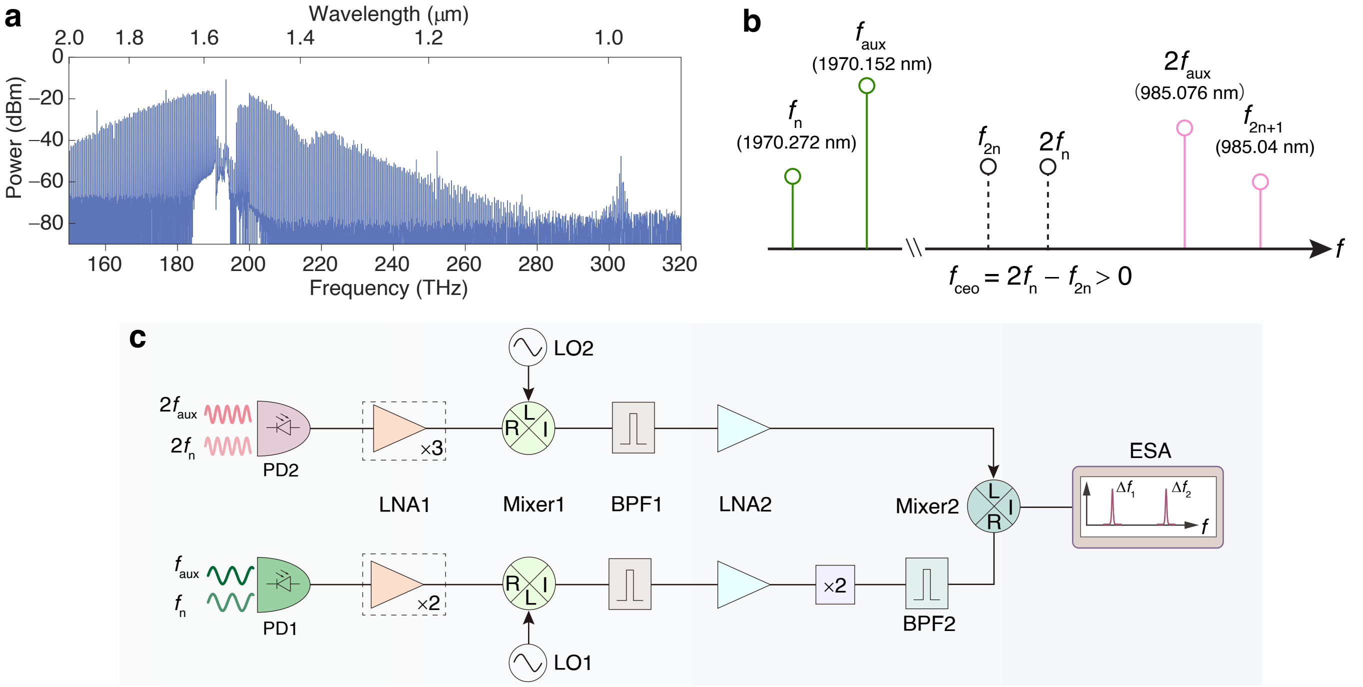

Supplementary Figure 6. **Implementation of f–$2f$ interferometry**. **a** Octave soliton spectrum after suppressing the pump by a FBG (see full spectrum in Fig. 4b of the main text). **b**. Sketch of the involved frequencies (monitored by the OSAs) around the f and $2f$ bands for the $f_{\rm ceo}$ measurement. **c**. Illustration of the experimental setup for electronically accessing the $f_{\rm ceo}$ based on a down-conversion frequency process. PD1: 830–2150 nm, 0–12.5 GHz; PD2: 800–1700 nm, 0–26 GHz; LNA1: 6–18 GHz, gain ≈ 27 dB; LNA2: 10–800 MHz, gain ≈ 60 dB; Mixer1: L/R port (7.5–20 GHz), I port (0–7.5 GHz); BPF1: 20–1000 MHz; RF doubler: 10–1000 MHz; BPF2: 1.5–2 GHz; Mixer2: L/R port (1–2700 MHz), I port (1–2000 MHz).

In order to expand the electronic accessing range of the f_{ceo} beatnote, we leverage a down-conversion process as sketched in Supplementary Fig. 6c, where incident lights in the f and $2f$ paths respectively beat at high-speed photodetectors (PD1 and PD2), and the generated beatnotes are boosted by cascaded low-noise amplifiers (LNA1) before sent into the RF Mixers (Mixer1) for producing down-converted frequency signals below 1 GHz. After bandpass filtering (BPF1, 20–1000 MHz), the down-converted beatnotes are boosted by high-gain LNA2s for driving a RF doubler and a Mixer2 in the f and $2f$ paths, respectively. The frequency-doubled signal is selected by another bandpass filter (BPF2) and the mixing frequency signals in the Mixer2 are monitored by an ESA (20 Hz–26.5 GHz).

The Mixers are driven by two tuned local oscillators (LO1 and LO2) covering a frequency span of 0–40 GHz (f_{LO1}) and 0–20 GHz (f_{LO2}), respectively. In the experiment, we chose f_{LO1} and f_{LO2} to be larger than δ_1 and δ_2. As a result, the output frequencies from the Mixer2 read:

$$\begin{aligned}\Delta f_1 &= 2f_{\mathrm{LO1}} - f_{\mathrm{LO2}} - (2\delta_1 - \delta_2)\\ \Delta f_2 &= 2f_{\mathrm{LO1}} + f_{\mathrm{LO2}} - (2\delta_1 + \delta_2)\end{aligned} \tag{4}$$

It is noteable that this scheme allows for an accessible f–$2f$ beatnote (that is $2\delta_1 + \delta_2$) up to $2f_{\mathrm{LO1}} + f_{\mathrm{LO2}}$, which is 100 GHz in our apparatus.

IV. SUPPLEMENTARY REFERENCES

[1] M. Zhang, C. Wang, R. Cheng, A. Shams-Ansarand, and M. Loncar. "Monolithic ultra-high-Q lithium niobate microring resonator". Optica **7**(12), 1536–1537 (2017).

[2] Z. Gong, X. Liu, Y. Xu, and H. X. Tang. "Near-octave lithium niobate soliton microcomb". Optica **7**(10), 1275–1278 (2020).

[3] T. Herr, V. Brasch, J. D. Jost, I. Mirgorodskiy, G. Lihachev, M. L. Gorodetsky, and T. J. Kippenberg. "Mode Spectrum and Temporal Soliton Formation in Optical Microresonators". Phys. Rev. Lett. **113**, 123901 (2014).

[4] X. Liu, C. Sun, B. Xiong, L. Wang, J. Wang, Y. Han, Z. Hao, H. Li, Y. Luo, J. Yan, T. Wei, Y. Zhang, and J. Wang. "Integrated continuous-wave aluminum nitride Raman laser". Optica **4**(8), 893–896 (2017).

[5] T. Herr, V. Brasch, J. D. Jost, C. Y. Wang, N. M. Kondratiev, M. L. Gorodetsky, and T. J. Kippenberg. "Temporal solitons in optical microresonators". Nat. Photon. **8**, 145—152 (2014).

[6] Z. Gong, A. Bruch, M. Shen, X. Guo, H. Jung, L. Fan, X. Liu, L. Zhang, J. Wang, J. Li, J. Yan, and H. X. Tang. "High-fidelity cavity soliton generation in crystalline AlN micro-ring resonators". Opt. Lett. **43**(18), 4366-4369 (2018).

[7] X. Liu, C. Sun, B. Xiong, L. Wang, J. Wang, Y. Han, Z. Hao, H. Li, Y. Luo, J. Yan, T. Wei, Y. Zhang, and J. Wang. "Generation of multiple near-visible comb lines in an AlN microring via $\chi^{(2)}$ and $\chi^{(3)}$ optical nonlinearities". Appl. Phys. Lett. **113**(17), 171106 (2018).

[8] A. W. Bruch, X. Liu, X. Guo, J. B. Surya, Z. Gong, L. Zhang, J. Wang, J. Yan, and H. X. Tang. "17,000%/w second-harmonic conversion efficiency in single-crystalline aluminum nitride microresonators". Appl. Phys. Lett. **113**(13), 131102 (2018).
